# Design and Actuation of a Fabric-Based Worm-Like Robot †

**DOI:** 10.3390/biomimetics4010013

**Published:** 2019-02-06

**Authors:** Akhil Kandhari, Anna Mehringer, Hillel J. Chiel, Roger D. Quinn, Kathryn A. Daltorio

**Affiliations:** 1 Mechanical and Aerospace Engineering, Case Western Reserve University, Cleveland, OH 44106, USA; agm72@case.edu (A.M.); rdq@case.edu (R.D.Q.); 2 Departments of Biology, Neurosciences and Biomedical Engineering, Case Western Reserve University, Cleveland, OH 44106, USA; hjc@case.edu

**Keywords:** soft robotics, worm-like robot, fabric-based robot

## Abstract

Soft-bodied animals, such as earthworms, are capable of contorting their body to squeeze through narrow spaces, create or enlarge burrows, and move on uneven ground. In many applications such as search and rescue, inspection of pipes and medical procedures, it may be useful to have a hollow-bodied robot with skin separating inside and outside. Textiles can be key to such skins. Inspired by earthworms, we developed two new robots: FabricWorm and MiniFabricWorm. We explored the application of fabric in soft robotics and how textile can be integrated along with other structural elements, such as three-dimensional (3D) printed parts, linear springs, and flexible nylon tubes. The structure of FabricWorm consists of one third the number of rigid pieces as compared to its predecessor Compliant Modular Mesh Worm-Steering (CMMWorm-S), while the structure of MiniFabricWorm consists of no rigid components. This article presents the design of such a mesh and its limitations in terms of structural softness. We experimentally measured the stiffness properties of these robots and compared them directly to its predecessors. FabricWorm and MiniFabricWorm are capable of peristaltic locomotion with a maximum speed of 33 cm/min (0.49 body-lengths/min) and 13.8 cm/min (0.25 body-lengths/min), respectively.

## 1. Introduction

The growing field of soft robotics demonstrates the mechanical and algorithmic advantages of using compliant materials [1,2]. Low modulus polymers allow robots to bend in ways that conventional robots cannot [3]. This enables robots to better accommodate human interaction and perform delicate operations. Furthermore, soft materials better mimic biology, allowing robotic platforms to imitate animals such as octopi [1,4,5], caterpillars [6], snails [7], and earthworms [8,9,10,11,12,13,14,15], as presented in this article.

Textiles can be key materials for future soft robots. A wide variety of fabrics are available that can provide advantageous weight, flexibility, strength, and cost properties [16]. Fabric has been used in wearable robotic devices that could provide active assistance during walking [17,18], such as robotic gloves for hand assistive applications [19,20], or have sensors embedded for monitoring electrophysiological information from the human body [21,22,23,24,25]. Fabric embedded with shape memory materials could be useful for an active joint stability brace on human fingers because it can change stiffness [26]. Pneumatic actuators embedded with fabric have been used to turn inanimate objects into multifunctional robots [27]. New approaches to replacing structural elements with textiles will be critical for these and other applications.

In this paper, we will show how fabric can replace traditional structural and compliant elements in an earthworm-like robot to achieve comparable performance with fewer mechanical parts. We directly compare a nonfabric-based design with a fabric-based design, demonstrating peristaltic locomotion on substrates with different coefficients of static friction.

The robots we present take their inspiration from earthworms. Earthworms are particularly skilled at navigating through confined spaces, complying with their surroundings, and burrowing. Earthworms use their segmented body to locomote. Each segment of an earthworm consists of a set of longitudinal and circumferential muscles. The hydrostatic coupling [28] allows the segment to extend longitudinally while contracting in diameter and vice versa. Earthworms travel by means of waves of muscular contractions called peristalsis [29], which causes the segments of the body to sequentially elongate and shorten in length, generating locomotion in the direction opposite to that of the muscular contractions. During peristalsis, the circumferentially expanded segments anchor the body allowing contracting segments to advance. Mimicking this type of locomotion on a robotic platform would be valuable in navigating constrained environments.

**Figure 1:**
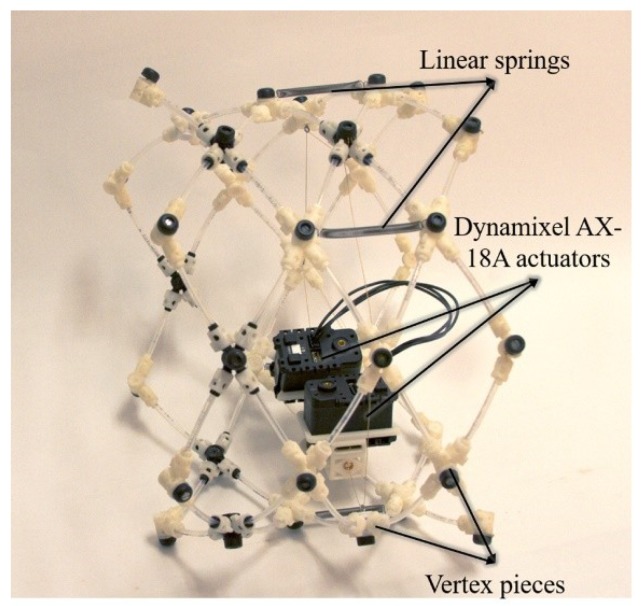
Our previous worm-like robots’ segments mimic worm segments with a cylindrical mesh held together with 3D printed vertex pieces and linear springs. Single segment of Compliant Modular Mesh Worm-Steering robot (CMMWorm-S) which has two AX-18A actuators and 22 3D printed rigid components (vertex pieces). The vertex pieces are connected using flexible nylon tubes. The linear springs placed along the length of the segment passively return the segment to its maximum diameter. The CMMWorm-S has redesigned 3D printed vertex pieces which replace the commercial quick-connect fittings used to connect tubes in the first iteration [10]. The 3D printed components are printed in acrylonitrile butadiene styrene on a Stratasys Fortus 400 mc FDM (fused deposition modeling) machine with 0.245 mm slice height.

Many worm-like robots have been constructed [8,9,10,11,30]. The Compliant Modular Mesh Worm robots (CMMWorm-Original and CMMWorm-Steering (CMMWorm-S; Figure 1)) developed by our group [9,10] are modular multisegmented, cable actuated soft robots. Both CMMWorm robots have a mesh structure held together with three-dimensional (3D) printed rigid components referred to as vertex pieces. Vertex pieces are connected with short links of flexible nylon tubes that allow relative rotation and prevent relative translation. A cable running along the circumference of each segment is actuated by either one (CMMWorm-Original) or two (CMMWorm-S) servomotors, similar to the circumferential muscle of an earthworm’s segment. As an actuator rotates, it spools in cable, thereby contracting the diameter of the segment. Linear springs are placed along the length of the segment that extend as the segment contracts in diameter. As the actuator spools out cable, the springs passively return the segment to its maximum diameter state, based on the amount of cable spooled out, similar to the longitudinal muscles of an earthworm’s segment.

Here, we present the structure of two fabric-based worm-like robots. Like earthworms and our prior robot CMMWorm, the body structures achieve locomotion by coupling radial expansion with longitudinal contraction. The FabricWorm, with a maximum diameter of 21 cm, has a mesh structure consisting of one third the number of rigid components (vertex pieces), as compared to CMMWorm, and nylon tubes encased within two layers of stretchable fabric. The MiniFabricWorm, with a maximum diameter of 12.5 cm, has a mesh structure consisting of nylon tubes encased within two layers of stretchable fabric and no rigid vertex pieces. This article describes the diameter–length coupling ratio, stiffness properties, and speed achieved by the two robots on different substrates with different coefficients of static friction. We report that the maximum speed achieved by FabricWorm is 33 cm/min (0.49 body-lengths/min) and that of MiniFabricWorm is 13.8 cm/min (0.25 body-lengths/min). The highest speed for both robots was achieved on linoleum tiles with a coefficient of static friction of 0.36 for MiniFabricWorm and 0.43 for FabricWorm. We also compare the performance of these robots to their nonfabric counterparts, which to the best of our knowledge is unique to the present work.

## 2. Robot Design

The design goal of this work is to reduce the number of rigid components in our prior worm-like robots (Softworm [8], Compliant Modular Mesh Worm Robot-Original [9], and Compliant Modular Mesh Worm Robot-Steering [10]). These rigid components limit the deformability of the robots. Our first fabric-based robot, FabricWorm, eliminated many of the 3D printed vertex pieces that held the mesh together and the linear springs. Our second version, MiniFabricWorm, is smaller and eliminated all the 3D printed vertex pieces of the mesh, leaving only the actuators as rigid components.

In both versions, two layers of stretchable fabric are sewn together around flexible tubes that intersect in a mesh pattern of rhombuses. The fabric compliance provides the spring return force that counters the cable actuation, eliminating the need for springs. The sleeves created by the fabric hold the mesh together, reducing (in the case of FabricWorm) and eliminating (in the case of MiniFabric Worm) the need for the pin joints created by the vertex pieces. The flexibility of the fabric allows large elastic deformation of the mesh structure, where an increase in length is coupled with a decrease in diameter (similar to the hydrostatic coupling observed in earthworms).

**Figure 2:**
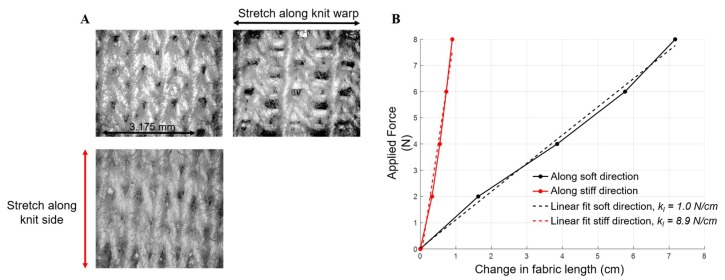
Stiffness of cotton fabric used on the fabric robots. (**A**) Structure of the chosen fabric, captured by a microscope with 100× magnification. Top left is the fabric in its undeformed state, top right shows stretch along the knit warp side (*k_l_* = 1 N/cm), and bottom panel shows stretch along the knit side (*k_l_* = 8.9 N/cm). (**B**) Change in length of fabric as a force is applied along the knit side and knit warp. The slope of the linear fit for the two curves is the stiffness of the material along orthogonal directions. Dimension of fabric tested was 2.54 cm × 2.54 cm. *k_l_*: Longitudinal stiffness.

Fabric selection is critical. Fabric elasticity can come either from elastic composite fibers or from the way the fabric combines those fibers; for example, knits permit greater stretch than weaves [31,32]. For FabricWorm and MiniFabricWorm, we used a knitted cotton fabric that was made of 97% cotton and 3% spandex (Jo-Ann Fabrics, Hudson, OH, USA). We used a knitted cotton fabric, since these can achieve large, recoverable deformations with a strain of ≈300% [31]. The selected fabric has anisotropic stiffness properties (Figure 2), which we used to provide recovery forces. Two pieces of fabric stitched together have a stiffness of 9 N/cm along its stiff direction (referred to as knit side) and 1 N/cm along its soft direction (referred to as knit warp). Fabric has negligible bending stiffness, but the stretchability can be directly compared to linear spring stiffness (range between 0.9 and 3.8 N/cm for the CMMWorm robots). The stiffer crosswise direction was aligned with the circumferential direction of the body, as the fabric provides the passive restoring force to return segments to the maximum (unactuated) diameter. This eliminates the need for longitudinal springs used in previous iterations [9,10].

The mesh of flexible tubes is integrated into the fabric to give the structure a compliant worm-like shape. Without fabric, the flexible tubes alone are not capable of returning to the maximum diameter state after circumferential deformation. Conversely, without the tubes, the fabric alone does not hold a cylindrical shape under gravity, nor can it be contracted uniformly. For FabricWorm, tubes of length 18.5 cm are connected using 3D printed rigid vertex pieces that were used in CMMWorm [9]. Nylon tubes with different bending stiffness properties were used to test FabricWorm. A stiffer tube had wall thickness of 0.66 mm with bending stiffness (*k_b_* = *EI*), where *E* is the Young’s modulus and *I* is the area moment of inertia, of 14.4 N·cm^2^ and a softer tube had wall thickness of 0.4 mm with *k_b_* of 2.8 N·cm^2^. On the other hand, MiniFabricWorm lacks any rigid components in its structure (i.e., the structure does not consist of any vertex pieces). The entire structure is composed of tubes spiraled along the entire length of the robot embedded within the fabric. The tube used had a wall thickness of 0.635 mm and a bending stiffness of 32.64 N·cm^2^. The assembly of both robots is discussed below.

### 2.1. Fabric Worm

**Figure 3:**
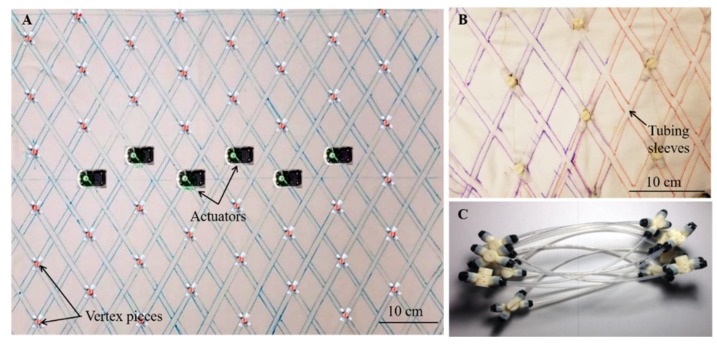
Assembly of the fabric-based worm robots. (**A**) The lines drawn on one layer of fabric serve as a guide for sewing sleeves for the tubing to pass through. The pink dots show where the vertex pieces will be placed and the placement of the actuators for a six-segment robot is marked. (**B**) The tubes are fed through the sleeves and the actuation cable is fed through the vertex pieces. The vertex pieces are also encased within the two layers of fabric. (**C**) Tubing used is shown without the fabric. The vertex pieces shown in this figure are from the first iteration of Compliant Modular Mesh worm robot [9], whereas Figure 1 shows the second iteration of the Compliant Modular Mesh Worm robot (CMMWorm-S) [10].

Two 80 cm × 70 cm rectangles were cut out of the fabric, with the 70 cm length in the direction of the stretch. A template using tubes and vertex pieces was assembled and placed on the fabric to create an outline of sleeves through which the tubes would pass [33]. The two fabric pieces were then sewed along these lines with all-purpose thread on an Opal 650 sewing machine (Husqvarna Viking) using a straight stitch, while making sure no strain was introduced during this process. This resulted in sleeves to encase the tubing (Figure 3) [34]. Once the tubes are inserted inside the sewn sleeves, the structure was rolled into a cylindrical shape and the two edges of the fabric were joined using sew-on snaps (Jo-Ann Fabrics) on the top. The sew-on snaps allow for easy access to inner components (vertex pieces or cables) for assembly and repair purposes.

The rhombus pattern of the mesh causes the length-width coupling that creates a change in diameter in response to a change in length. The included angle of the rhombus (the sewing angle of the sleeves) is based on the limits of the rigid vertex pieces, allowing maximum range of motion. The final assembly of FabricWorm consists of one third the number of rigid pieces (48 as compared to 132) when compared to its predecessor Compliant Modular Mesh Worm robot. Each segment consists of six rigid components, one actuator mount, and five vertex pieces. The actuator mount houses the Dynamixel MX-64T actuator (Robotis, Lake Forest, CA, USA). A cable running along the circumference of the segment is actuated by the motor, allowing circumferential contraction (causing longitudinal extension). As the cable is unspooled, the fabric around the mesh passively expands the diameter of the segment, based on the amount of cable unspooled until it reaches its maximum possible diameter (rest state). A sequential circumferential contraction and expansion of connected segments allows the robot to locomote in a direction opposite to the direction of the contraction–extension wave.

**Figure 4:**
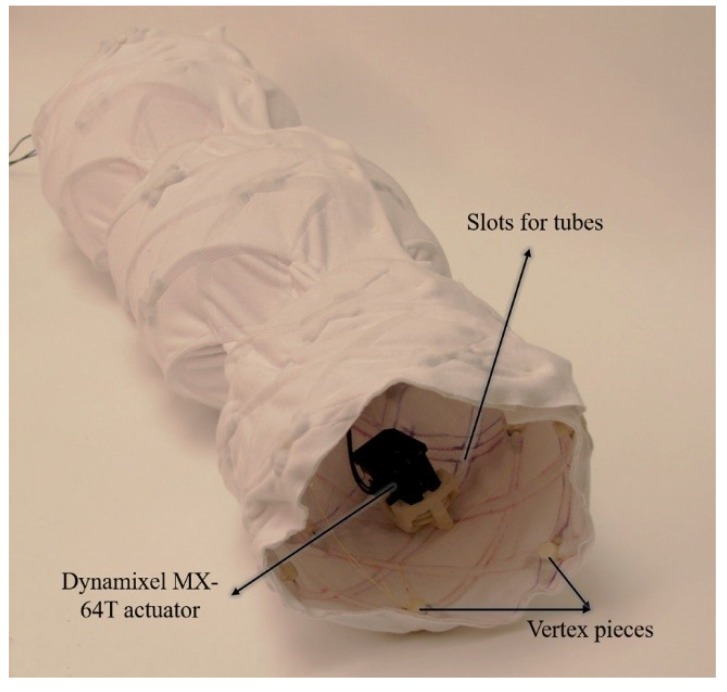
FabricWorm during a peristaltic wave. The various components of the structure are labelled. The sleeves through which the tubes pass can be seen on the inside of the fabric. The total length of FabricWorm in its rest state is 67 cm.

**Figure 5:**
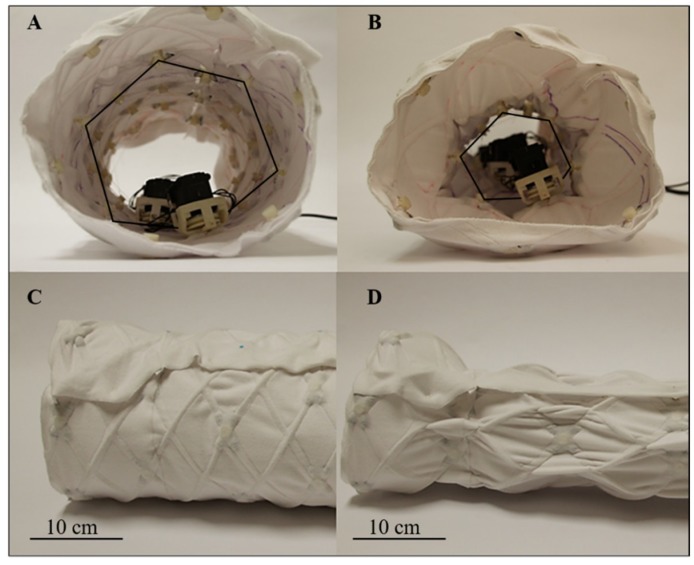
FabricWorm in different actuation phases. (**A**) Front view of FabricWorm fully expanded. The actuation cable is highlighted (black hexagon drawn over the cable for visibility). (**B**) As the actuation cable (smaller black hexagon) is spooled-in, the robot contracts in diameter. (**C**) Side view of fully expanded FabricWorm. (**D**) Side view of fully contracted FabricWorm.

The fully assembled FabricWorm (Figure 4) [34] has six segments with six actuators and a total of 48 3D printed components. The total robot weighs 1.61 kg and has a rest length of 67 cm. The maximum diameter is 21 cm and the robot is capable of deforming to 60% of its maximum diameter (Figure 5) [34]. The fabric worm body is deformable and is capable of being bent in a semicircular shape.

### 2.2. MiniFabricWorm

**Figure 6:**
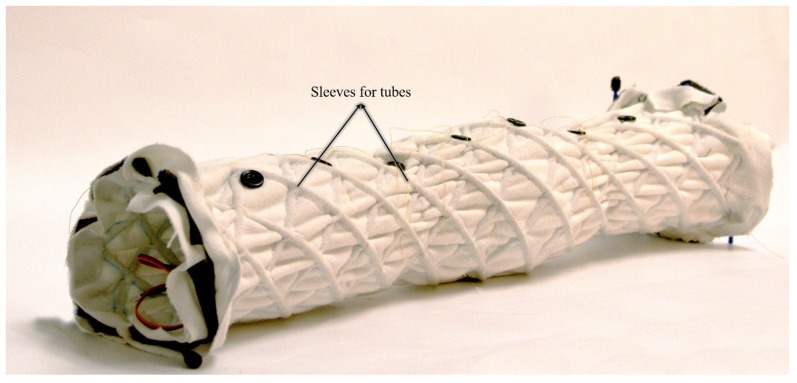
The MiniFabricWorm consists of no rigid components in the structure. The sleeves through which tubes pass are visible. The length of MiniFabricWorm in its rest state is 55 cm.

The MiniFabricWorm (Figure 6) builds on the design of the FabricWorm, but simplifies it. In contrast, its maximum diameter is smaller compared to FabricWorm (12.5 cm as compared to 21 cm). Miniaturization of FabricWorm was limited by the presence of 3D printed rigid pieces in the structure. Thus, MiniFabricWorm does not include any rigid pieces in the structure. The design of MiniFabricWorm includes two layers of fabric sewn together just as with FabricWorm to create sleeves for tubing. Instead of having sections of tubes connected to vertex pieces, there are 12 longer tubes helically fed throughout the entire length of the robot. The assembly of MiniFabricWorm is the same as FabricWorm (Figure 3), without the use of vertex pieces to join sections of tubing. The intersections of the tubing in the sewn fabric sleeves replace the vertex pieces. Each segment consists of only one rigid piece: the actuator mount. Actuator mounts are held inside the fabric integrated mesh by being sewed through the fabric.

The MiniFabricWorm uses smaller Dynamixel AX-18A actuators (Robotis) that are housed within the actuator mounts. Cables attached to the actuator are sewn through the fabric across the circumference of the segment and secured to buttons that are sewn on the top of the body. The working principle is the same as FabricWorm: as the cable is spooled in, the segment decreases in diameter and, as the cable is spooled out, the fabric allows circumferential expansion, based on the amount of cable spooled out.

The MiniFabricWorm uses tubes of higher bending stiffness compared to the stiffest tubes used in FabricWorm (bending stiffness of 32.64 N·cm^2^ compared to 14.4 N·cm^2^). The reason is that when tubes of lower bending stiffness were used, the robot failed to locomote due to the softness of its structure (i.e., the structure was too compliant and did not cause the robot to move). Replacing tubes within the mesh with higher bending stiffness tubes increased the bending stiffness and circumferential stiffness of the robot based on Kandhari et al. [10]. This allowed the robot to move.

MiniFabricWorm fully assembled is a five-segment worm-like robot with only one rigid component (actuator mount) within the structure. The robot weighs 580 g with a maximum diameter of 12.5 cm and minimum diameter of 10.2 cm. The total length of the robot in its rest state is 55 cm. The structural comparison of CMMWorm-S, FabricWorm, and MiniFabricWorm is summarized in Table 1.

The fabric worm robots, compared to their nonfabric counterparts [9,10], are much faster to fabricate and assemble due to the greatly reduced number of parts. It is much faster to sew together two pieces of fabric than to 3D print an additional 84 vertex pieces. Moreover, the manual assembly process is a significant cost and time factor. Fabric-based robots were faster to assemble because of the elimination of 14 (for FabricWorm) to 22 (for MiniFabricWorm) vertex pieces per segment. The fabric counterparts also do not require the attachment of discrete longitudinal springs. Furthermore, each vertex piece costs $1.70 at standard 3D printing rates of $0.6/cm^3^ at Case Western Reserve University’s public maker space think[box]. The fabric, which costs approximately $12/m^2^ at Jo-Ann Fabrics, is a cost-saving solution.

### 2.3. Electronics and Control

FabricWorm and MiniFabricWorm were actuated by Dynamixel MX-64T and Dynamixel AX-18A actuators, respectively. These actuators were connected to a single Robotis OpenCM9.04 microcontroller (Robotis). Programming of the microcontroller and data logging were performed over a universal serial bus (USB) to personal computer (PC) connection. We used our open source DynamixelQ library [35] for the OpenCM9.04 microcontroller that allows high-speed communication with AX and MX series Dynamixel actuators.

A time-based control scheme generates waves along the length of the robot to produce locomotion. Both FabricWorm and MiniFabricWorm used a 3 × 1 wave pattern (where 3 represents the number of segments per wave and 1 the number of waves along the body, following the convention by Horchler et al. [9] and Kandhari et al. [10]. At any given time, one segment is contracting in diameter and one is expanding, with an inactive spacer segment in between. After sufficient time has passed for contraction and retraction (1.8 s for FabricWorm and 0.9 s for MiniFabricWorm), allowing the contracting segment to achieve minimum diameter and the retracting segment to achieve maximum diameter, the wave shifts down the body, resulting in forward motion.

## 3. Results and Discussion

We empirically characterized the properties and resulting performance of both robots. For FabricWorm, two types of tubes with different bending stiffness properties were used to determine robot properties. We determined robot properties for both robots in terms of diameter–length coupling ratio, longitudinal stiffness, bending stiffness, and robot speed on substrates with different coefficients of static friction.

### 3.1. Diameter–Length Coupling Ratio

**Figure 7:**
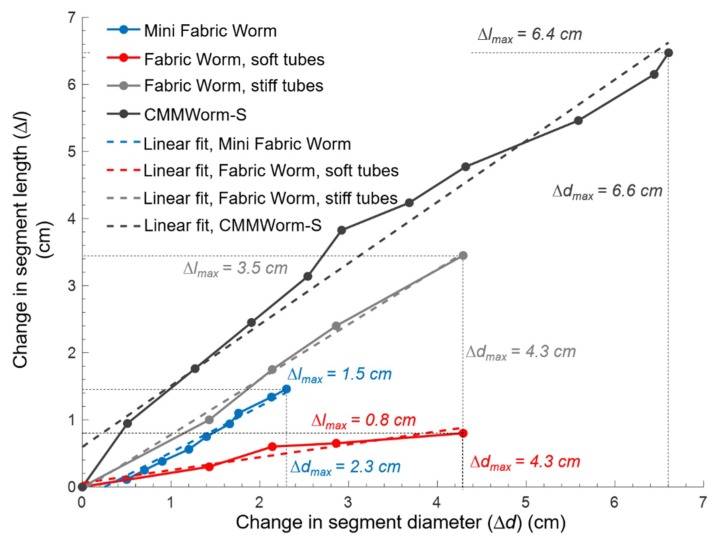
Coupling ratio, which is the relationship between the change in length of a segment and the change in diameter of a segment for MiniFabricWorm (blue line), FabricWorm with soft tubes (red line), and FabricWorm with stiff tubes (gray line). MiniFabricWorm (blue line) has a coupling ratio of 0.68, whereas FabricWorm with stiff tubes has a coupling ratio of 0.82. CMMWorm (black line) has the highest coupling ratio of 0.92. A large coupling ratio will result in better longitudinal extension allowing larger stroke lengths per peristaltic wave. CMMWorm is capable of contracting by 32% of its maximum diameter while extending by 37% of its initial segment length, whereas MiniFabricWorm and FabricWorm with stiff tubes both contract by 20% of their initial diameter and extend by 16% and 31% of their initial segment length, respectively.

To determine how change in diameter will be translated to a change in length, a coupling ratio was evaluated for both robots. This made it possible to understand how well the mesh can translate forces within the structure, allowing longitudinal extension and causing locomotion. If a change in diameter does not induce a sufficient change in length due to the structure being too soft or too stiff, the robot will not be able to move. In case of a fabric integrated mesh, it is important to see if the fabric allows simultaneous changes in length and diameter (Figure 7).

To measure the coupling ratio for FabricWorm, the front two segments were contracted and the change in diameter and length were noted for both versions of FabricWorm (with stiff and soft tubes). The change in length was divided by two for change in segment length. For MiniFabricWorm, all five segments were contracted and the change in diameter and length was noted. Length was divided by five for change in segment length. Measuring the coupling ratio of the robots is a quality indicator of how well the robot will move based on the structural components and stiffness.

In MiniFabricWorm, the coupling ratio was 0.68. The change in diameter for MiniFabricWorm could not exceed 2.3 cm, as the presence of actuator limits further contraction. In FabricWorm with stiff tubes, the coupling ratio for a single segment was 0.82, whereas with soft tubes it was 0.19. The low coupling ratio of 0.19 is due to the low bending stiffness of the tubing. During locomotion testing, FabricWorm units with these low bending stiffness tubes were unable to locomote, because as the segments were actuated, the tubes buckled and kinked instead of extending the segments in length. The compliance of the structure absorbs the actuation locally rather than transmitting force to the rest of the structure. Hence, all locomotion testing for FabricWorm was done with the stiff tubes. CMMWorm-S has the highest coupling ratio of 0.92. Although our goal was to make the robot as soft as possible, this data shows that there is a limit to how soft the structure can be for locomotion using this design.

### 3.2. Logitudinal Stiffness

**Figure 8:**
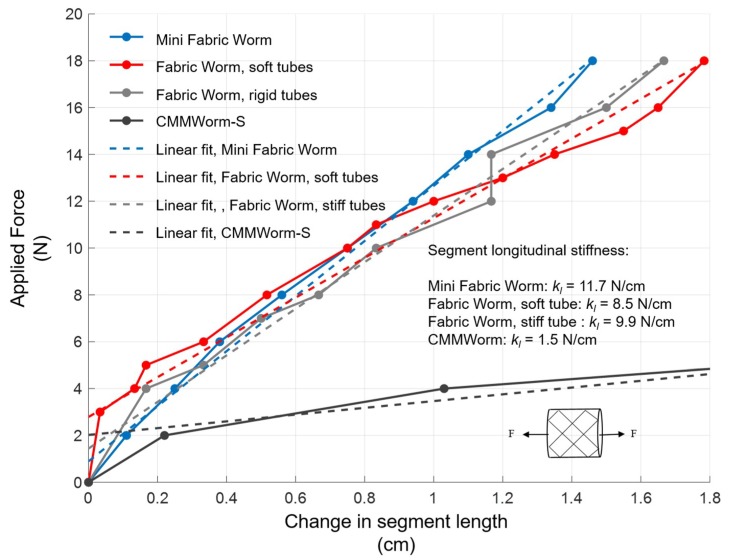
Change in length of a single segment when a force is applied along the length of the segment. The slope of the linear fit (dashed lines) of the curves is used to estimate the longitudinal stiffness of the segment. *k_l_*: Longitudinal stiffness; *F*: Force applied in the horizontal direction.

The longitudinal stiffness of the robot was measured by securing one end of the robot and applying incrementally increasing loads to the other end while measuring extension of the robot. The slope of the regression line between displacement and force is the measured longitudinal stiffness (Figure 8).

The stiffness of a segment of FabricWorm is 8.5 N/cm when the mesh consists of soft tubes and 9.9 N/cm when the mesh is composed of stiff tubes. For MiniFabricWorm, the longitudinal stiffness was 11.8 N/cm.

Figure 8 shows that the main factor determining the longitudinal stiffness of a segment is the stiffness of the fabric along its knit side, and thus all the FabricWorm stiffnesses are similar. The tubes, however, also add to the overall longitudinal stiffness; as a consequence, MiniFabricWorm, which had the tubes of the highest bending stiffness, had the highest longitudinal stiffness, followed by FabricWorm with stiff tubes and FabricWorm with soft tubes. FabricWorm with soft tubes exhibits uneven deformation beyond an extension of 1 cm as the tubes started to buckle. As the fabric is responsible for circumferential expansion of the segment on removal of the actuation force, a higher stiffness fabric will cause an increase in the longitudinal stiffness of the segment.

In comparison to CMMWorm-S, the stretched fabric makes the robot stiffer. That is because the stiffness of the fabric itself is stiffer than the springs used in the CMMWorm-S robot. The total spring stiffness on CMMWorm-S was 1.8 N/cm and the overall stiffness of the segment was 1.5 N/cm. Similarly, all the stiffnesses of the fabric worm robots are similar to the stiffnesses of the fabric; the small differences in these values are due to the different tubes used in the structure.

### 3.3. Bending Stiffness

**Figure 9:**
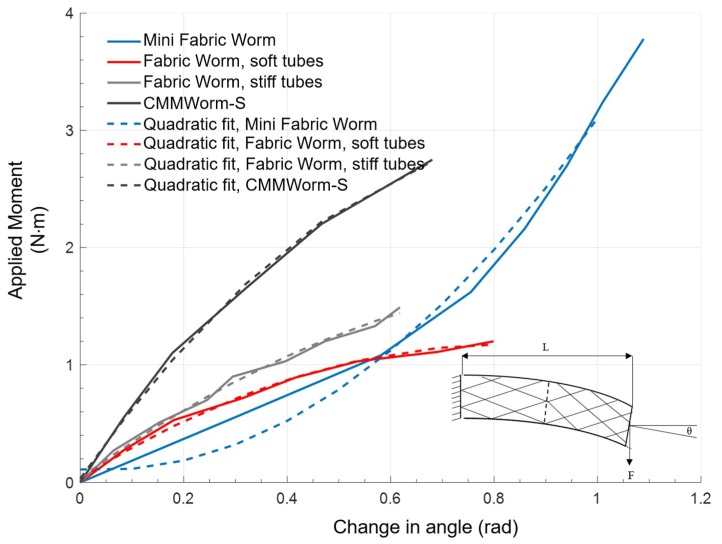
Change in angle of the robot as a moment is applied at the end of the robot. Both robots follow a nonlinear trend. The joint angle of the rhombus structure is an important factor causing the nonlinear trend. Joint angle decreases with increasing bending stiffness, thereby decreasing bending capability when rigid components are present. Moment that can be applied on MiniFabricWorm is larger compared to FabricWorm due to the absence of rigid components in the mesh. *L*: Length of robot being tested; *F*: Force applied at the end; *θ*: Angle by which the robot bends along the horizontal axis.

Bending stiffness of the robot provides a quantitative measure of the ability of a contracted segment to support itself between expanded anchoring segments. A peristaltic robot with a higher bending stiffness will be capable of lifting its adjacent contracted segments off the ground during longitudinal extension. As segments are lifted during locomotion, frictional resistance decreases compared to when segments are dragged along the substrate, thereby improving locomotion. Bending stiffness (Figure 9) determines the overall compliance of the structure and depends on the fabric, the tubes, the rigid components (if present), and the included angle of the rhombus patterns (i.e., a fully stretched robot has a lower bending stiffness compared to a fully compressed robot).

**Figure 10:**
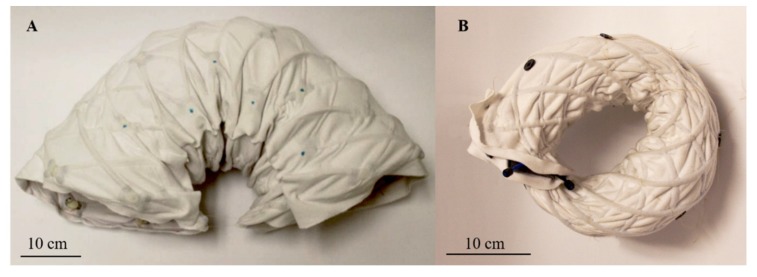
Images demonstrating the bending limits of these two robots. (**A**) FabricWorm bent in a semicircle (≈180°) due to the flexibility of the body. In this image, a string holds the ends together to maintain this position. The robot is not capable of bending any farther due to the presence of rigid components. (**B**) MiniFabricWorm in a circular configuration (≈360°). Due to the absence of rigid components in the mesh, the robot is capable of achieving this position. Further bending causes the actuator mounts within the robot to interfere with one another.

On increasing the applied moment, the change in angle follows a downward parabolic trend for FabricWorm and upward parabolic trend for MiniFabricWorm. This difference is due to the fact that FabricWorm has rigid components present in the structure and applying moment beyond a threshold might cause failure at these points of rigid contacts. Thus, a moment of up to only 1.4 N·m was applied for FabricWorm. However, due to the absence of any rigid vertex pieces in MiniFabricWorm, a larger moment could be applied without any points of failure (breaking of the rigid components) and a larger change in bending angle was observed. Overall, within its working region, CMMWorm has the highest bending stiffness followed by FabricWorm with stiff tubes then FabricWorm with soft tubes. Due to the absence of rigid components, MiniFabricWorm has the least bending stiffness in the operating range, making it the most compliant robot amongst the different iterations designed by our group (Figure 10) [8,9,10,34].

### 3.4. Robot Speed

**Figure 11:**
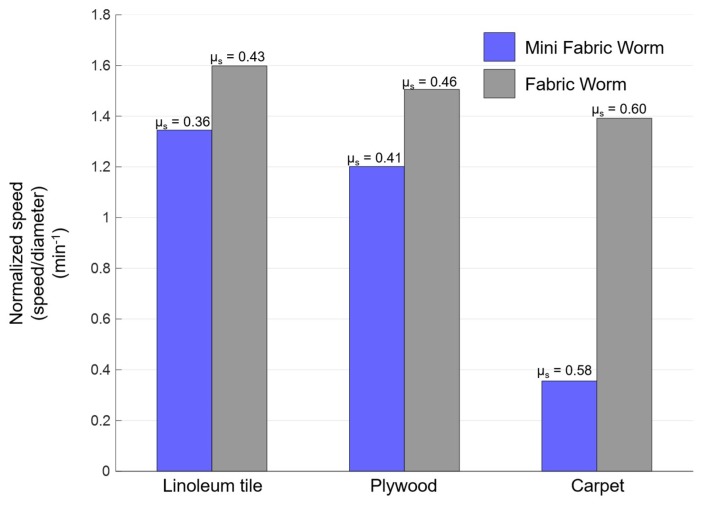
Robot speed normalized by diameter for FabricWorm with stiff tubes (gray) and MiniFabricWorm (blue). A video was taken from the sagittal view and Tracker software was used to measure the progress made over time for both robots on substrates with different coefficients of friction (indicated on top of each bar). The MiniFabricWorm robot was run three times over multiple wave cycles on linoleum tile and plywood, and twice on carpet. *μ_s_*: Coefficient of static friction.

Finally, we compared the speed of the robots in straight-line locomotion (Figure 11, Supplementary Videos S1 and S2). For FabricWorm, tests were performed using only stiff tubes, since it did not locomote with the soft tubes because of the structure being too compliant. MiniFabricWorm and FabricWorm were tested on surfaces with different coefficients of static friction (in ascending order): linoleum tile; plywood; and carpet. Videos from the sagittal view using an HD camera (Canon Vixia HF G30, 59.94 fps) were taken and Tracker Video Analysis software (Version 4.11.0, Open Source Physics, https://physlets.org/tracker/) was used to analyze the distance moved over multiple peristaltic cycles for both robots.

The robots achieved greater speeds with stiffer tubes and on substrates with the least coefficient of static friction (linoleum tiles). MiniFabricWorm achieved a maximum speed of 13.8 cm/min (0.25 body-lengths/min) on linoleum tile, followed by 11.7 cm/min (0.21 body-lengths/min) on plywood, and only 4 cm/min on carpet (0.07 body-lengths/min). FabricWorm achieved a maximum speed of 33 cm/min (0.49 body-lengths/min) on linoleum tile, 31.2 cm/min (0.46 body-lengths/min) on plywood, and 28.8 cm/min (0.43 body-lengths/min) on carpet. The limiting factor for speed is the change in diameter that causes a change in length. For MiniFabricWorm, the maximum change in diameter is 2.3 cm (18% of maximum diameter) and change in length is 0.8 cm. For FabricWorm, the maximum change in diameter is 4.3 cm (20% of maximum diameter) and change in length is 3.8 cm. MiniFabricWorm, due to its smaller diameter and change in segment length, has a smaller stroke length (extension of each segment during one cycle of peristaltic wave); thus, in Figure 11, we normalize the speed with maximum diameter.

Both robots experience slip in the forward and backward directions, and thus the speed decreases as the coefficient of friction increases. MiniFabricWorm on carpet makes little forward progress, because the carpet prevents the robot from moving forward: the fabric and carpet interact such that the robot gets stuck with each kernel of the carpet, thereby hindering forward locomotion. In contrast, for MiniFabricWorm, even without any rigid vertex pieces in the structure, the mesh-integrated fabric is capable of transmitting forces such that the segment contracts uniformly, thereby causing longitudinal extension. On removal of the actuation forces, the fabric allows for circumferential contraction that causes the robot to move forward.

## 4. Conclusions and Future Work

We have shown that a mesh can be integrated into a compliant fabric to form a structure that, when actuated, can mimic worm-like peristaltic locomotion. The fabric can secure the mesh so that it holds its shape during actuation. Moreover, the stretchable fabric can provide restoring forces, eliminating the need for other restoring springs. The stiffness of the fabric is nine times greater along its knit warp direction than along its knit side direction. We aligned the stiff side along the circumference of the robot so that it aids in passively returning the segment to its maximum diameter state as the actuation force is removed. As a result, compared to our prior robots, fewer rigid pieces are required. Specifically, FabricWorm uses 36% of the vertex pieces of CMMWorm, and MiniFabricWorm uses no vertex pieces at all in the mesh.

In the authors’ experience, reducing the number of rigid parts makes the mechanical design more robust. Our group has been improving the 3D printed connectors for modular worm robots for more than five years to achieve greater mechanical robustness, low weight, and low cost. The FabricWorm robots further advance the soft robotic paradigm: softer connections are less brittle, weigh less, and cost less.

Overall bending stiffness is key to performance. We previously demonstrated in Kandhari et al. [10] that reducing spring stiffness or using tubes with lower bending stiffness reduces stiffness properties. However, the structure is not capable of maintaining its cylindrical shape if tubes of lower bending stiffness are used. During locomotion, the softer tubes bend and kink easily which hinders locomotion because actuation forces are not transferred uniformly along the segment.

In Kandhari et al. [10] we developed design criteria for peristalsis. We demonstrated that the ratio of the expansion of a peristaltic robot to its contraction should be less than the capability of the segment to resist those changes. In other words, there exist limits on structural softness which depend on robot mass of the robot and the extent to which the robot segment can change in diameter and length. If the structure of the robot is too soft, all the actuation energy will be lost due to high compliance of the robot. In contrast, if the structure is too rigid, the robot will be unable to deform uniformly. Both these conditions will prohibit the robot from locomoting. Thus, for the case of MiniFabricWorm, we used nylon tubes with higher bending stiffness such that the robot could deform uniformly, allowing peristaltic locomotion.

When the performances of FabricWorm and MiniFabricWorm are compared with their predecessors, the advantages of fabric as a construction material are clear. Absence of rigid components reduces the overall bending stiffness of the two robots compared to the previous generation robots that our group had developed (approximate values: CMMWorm-S, 3.68 Nm/rad; FabricWorm, 2.3 Nm/rad; and MiniFabricWorm, 1.8 Nm/rad). Bending stiffness could be further decreased by increasing the length of the flexible tubes used. Pitch and spacing of the flexible tubes is inversely proportional to the bending stiffness. Integrating fabric in the mesh reduces the number of rigid components present in the structure that allows FabricWorm, and to a greater degree MiniFabricWorm, to bend and recover from large body bending.

Furthermore, we show that by selecting appropriate tube stiffness, locomotion can be achieved even with this much softer fabric mesh. The first key to locomotion is sufficient coupling ratio, (change in length vs. change in diameter). A low coupling ratio (such as 0.19 with soft tubes in FabricWorm) results in uneven deformations and poor locomotion. When stiffer tubes are used, the coupling ratio increases to 0.82, which permits locomotion. Even without the vertices, MiniFabricWorm achieves a coupling ratio of 0.68, allowing locomotion. This is comparable with coupling ratio of earthworms of 0.66 [9] and 0.92 [10] for CMMWorm robots.

We have previously hypothesized that low longitudinal stiffness can be valuable for eliminating slip if segments are imprecise [9], and have then shown that in practice high longitudinal stiffness increases the speed of worm-like mesh robots [10]. Here, we show that the longitudinal stiffness of the segment is highly dependent on the stiffness of the fabric that is aligned along the circumference of the segment. In our case, the stiffer, knit side was aligned along the circumference of the robot. Hence, the longitudinal stiffness values of the segment were comparable to the stiffness of the fabric.

We examined the speed of these two robots in straight-line locomotion on substrates with different coefficients of friction. Due to its higher coupling ratio, FabricWorm can achieve a maximum speed of 33 cm/min on linoleum tile, whereas MiniFabricWorm can achieve a maximum speed of 13.8 cm/min on the same substrate. Normalized by diameter, the speeds are comparable: 1.6 min^−1^ for FabricWorm as compared to 1.38 min^−1^ for MiniFabricWorm. These speeds are also similar to CMMWorm and CMMWorm-S, which have speeds of 25 cm/min (0.24 body-lengths/min) and 72 cm/min (0.92 body-lengths/min), respectively. The higher speed of CMMWorm-S as compared to FabricWorm is due to a higher coupling ratio and the use of faster actuators (97 rpm vs. 63 rpm at 12V). Actuator speed along with backward slip and compliance of structure are factors that limit robot speed [9].

This design enables future worm-like robots to utilize other advantages of fabric skin. The fabric skin protects the interior from debris. The skin is a surface upon which friction-altering surface treatments (e.g., anisotropic friction worm-like setae) can be affixed. Fabric-integrated mesh is light, highly flexible, and cheap to manufacture. The robot breaks less frequently, as compared to its predecessors, largely due to the reduction of breakable components. However, inherent limitations such as maximum strain before rupture, hysteresis, toughness, and fatigue still need to be better understood in future work [36]. Fabric skins may help traverse rough surfaces by deflecting entanglements or they may catch or tear on protrusions. In future work, fabric with self-healing properties [37], water resistance, and embedded sensing and actuation could be especially valuable for worm robots.

To move toward understanding locomotion where the robot has to execute sharper turns, squeeze through narrow constraints, burrow or travel on delicate surfaces, future versions of the robot will have more and potentially different types of actuation. Turning is essential, and will require left and right actuation. We have previously shown that low bending stiffness is correlated with slower turning [10]. However, eliminating slip in turning may permit better turning with lower bending stiffness [38]. Structural stiffness and actuation can work together to exert radial and axial forces for constrained space applications like burrowing. Novel actuation can change weight distribution and allow for greater deformability. In all these applications, the characterizations provided here can provide baseline comparisons for future designs.

## Figures and Tables

**Table 1:** Summary of structural comparison between CMMWorm-S, FabricWorm, and MiniFabricWorm.

	CMMWorm-S	FabricWorm	MiniFabricWorm
Mesh mass (g) ^1^	650	854	305
Number of rigid pieces in structure	132	48	0
Number of segments	6	6	5
Largest diameter (cm)	21	21	12.5
Total contracted length (cm)	103	67	55

^1^ Mass of entire robot’s mesh without actuators.

## Supplementary Materials

The following are available online at https://www.mdpi.com/2313-7673/4/1/13/s1, Video S1: FabricWorm locomotion on laminate top desk, Video S2: MiniFabricWorm locomotion on linoleum tile.
